# Catalytic DNA Polymerization Can Be Expedited by Active Product Release**

**DOI:** 10.1002/anie.202114581

**Published:** 2022-04-20

**Authors:** Pepijn G. Moerman, Momcilo Gavrilov, Taekjip Ha, Rebecca Schulman

**Affiliations:** ^1^ Chemical and Biomolecular Engineering Johns Hopkins University Baltimore MD 21218 USA; ^2^ Biophysics & Biophysical Chemistry Johns Hopkins University Baltimore MD 21205 USA; ^3^ Biomedical Engineering Johns Hopkins University Baltimore MD 21218 USA; ^4^ Howard Hughes Medical Institute Baltimore MD 21205 USA; ^5^ Chemistry Johns Hopkins University Baltimore MD 21218 USA; ^6^ Computer Science Johns Hopkins University Baltimore MD 21218 USA

**Keywords:** Active Processes, Biophysics, Catalysis, DNA Nanotechnology, Enzyme Catalysis, Kinetics

## Abstract

The sequence‐specific hybridization of DNA facilitates its use as a building block for designer nanoscale structures and reaction networks that perform computations. However, the strong binding energy of Watson–Crick base pairing that underlies this specificity also causes the DNA dehybridization rate to depend sensitively on sequence length and temperature. This strong dependency imposes stringent constraints on the design of multi‐step DNA reactions. Here we show how an ATP‐dependent helicase, Rep‐X, can drive specific dehybridization reactions at rates independent of sequence length, removing the constraints of equilibrium on DNA hybridization and dehybridization. To illustrate how this new capacity can speed up designed DNA reaction networks, we show that Rep‐X extends the range of conditions where the primer exchange reaction, which catalytically adds a domain provided by a hairpin template to a DNA substrate, proceeds rapidly.

## Graphical abstract

The rate of the primer exchange reaction is a strongly peaked function of the binding strength between the reactant and catalyst strands. This trend holds for many catalytic reactions and is known as Sabatier's principle. A helicase that consumes ATP to selectively remove the products from the catalysts can speed up the reaction in the strong‐binding regime and reduce the dependency of the catalytic rate on the binding strength.

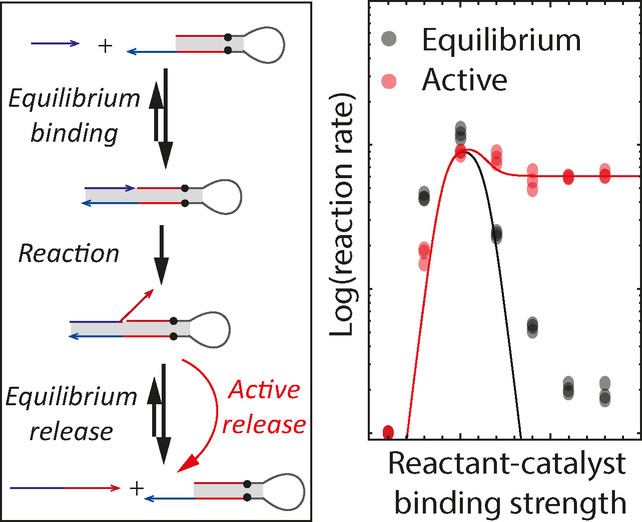

## Introduction

Sequence complementarity is the central design rule for building nanostructures and reaction networks from DNA.[^1^, ^2^] It enables DNA computers to recognize and report disease‐related RNAs among a slew of native oligonucleotides.[^3^, ^4^] It guides thousands of short strands simultaneously to their intended positions in two‐ and three‐dimensional structures,[^5^, ^6^] some of which can be reconfigured in response to DNA signals[^7^, ^8^] or pH changes.[^9^, ^10^] And it makes possible complex computations that take DNA strands as inputs and produce different DNA strands as outputs.[^11^, ^12^, ^18^] The binding specificity of oligonucleotides that makes these applications possible comes from the strong Watson–Crick base pairing: under standard conditions each pair contributes $1-4\;k_{B}T$ ,^[14]^ so that a strand strongly favors binding to its full complement over a spurious target with as little as one mismatch.^[16]^ The precise hybridization energy is sequence dependent and G−C pairs contribute approximately twice as much as A−T pairs as quantified in the nearest‐neighbor model.^[15]^

**Figure 1:**
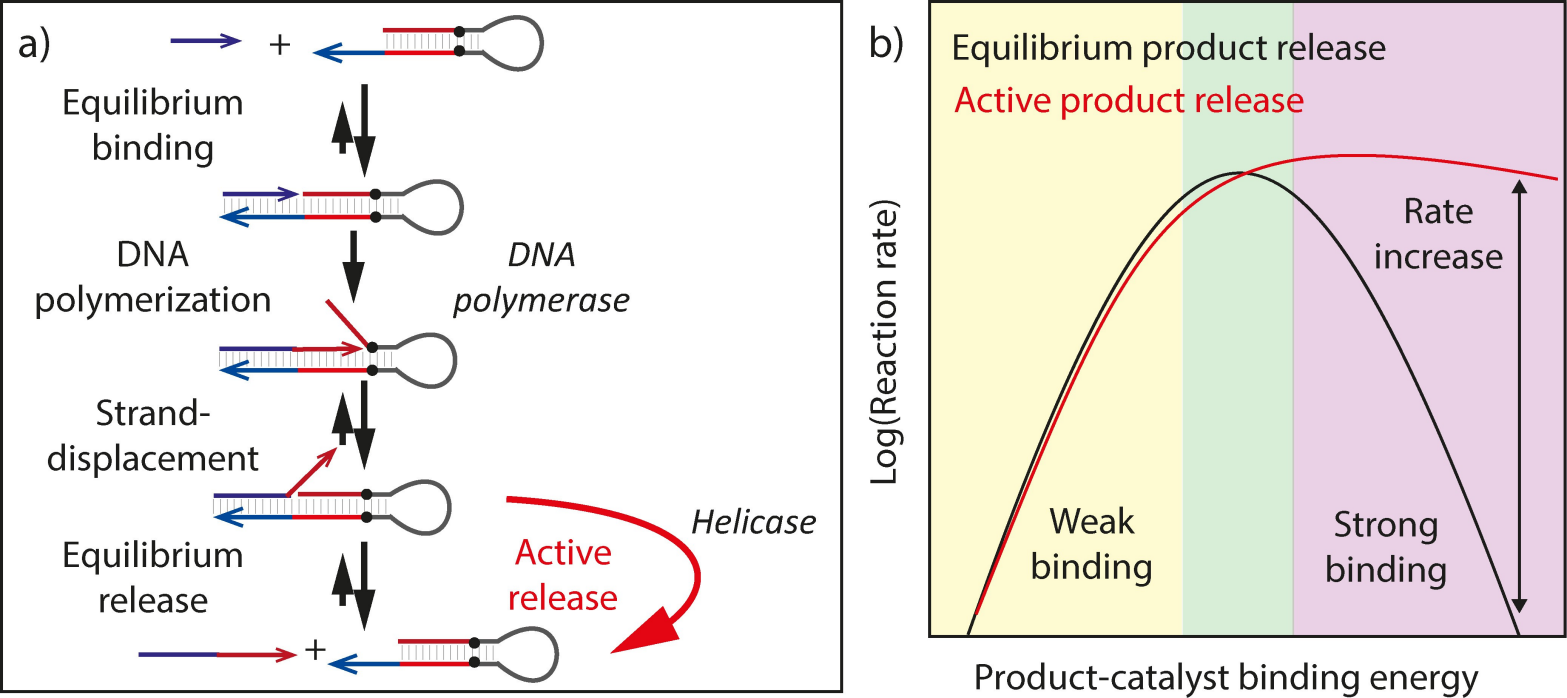
a) The primer exchange reaction involves two competing equilibrium hybridization steps: the reactant binding to the catalyst and the product binding to the catalyst. A helicase can dissipatively and selectively remove product from the catalyst, freeing the catalyst to bind to new reactant, and thereby expedite the reaction. b) A “volcano plot” captures the peaked reaction rate as a function of binding energy that is typical for catalytic reactions. Both weakly and strongly binding catalysts are ineffective: in the weak‐binding regime because no reactant is adsorbed and in the strong‐binding regime because no product is released. Only at intermediate binding energy does the reaction proceed rapidly. Active removal of the product could prevent catalyst poisoning and expedites the reaction in the strong‐binding regime.

Yet this strong dependence of the binding energy on oligonucleotide length can also be an Achilles’ heel in designing multi‐step reactions or reaction cascades. In such processes an individual sequence domain can participate in multiple reaction steps in which it has different functions (e.g. Figure 1a). These different steps may require conflicting binding and unbinding rates. For example, a long domain may provide the binding energy required to speed up the formation of one complex by stabilizing it, but then slow down a reaction elsewhere in the network that requires a high off‐rate. This conflict creates an upper limit on the effective rate of a multi‐step reaction, which can only be achieved at a optimal domain length and temperature. Consequently, many DNA reaction networks operate on timescales of hours.[^17^, ^18^, ^21^] The constraint that on‐ and off‐rates are coupled is a consequence of thermodynamic equilibrium: The upper limit on reaction rates is generic to any multi‐step, reversible chemical processes. In heterogeneous catalysis, it is known as Sabatier's principle,^[22]^ which states that reactions only proceed if substrate–catalyst binding is not too weak, but product‐catalyst binding not so strong that it poisons the catalyst (Figure 1b). Addressing this fundamental limit on the composite rate of multi‐step reactions requires energy input to subvert equilibrium.

Here we ask how an exergonic reaction can be used to decouple the off‐rates of DNA hybridization reactions that involve the same binding domain and thus reduce the dependency of the process's rate on the binding strengths. We use DNA helicases—a class of ATP‐dependent proteins that separate double‐stranded DNA into its single‐stranded components—to couple ATP hydrolysis to DNA unwinding. In vivo, helicases unwind parts of long double‐stranded DNA whose rates of dehybridization would otherwise be negligible to prepare genomic DNA for replication by exposing a template strand. We ask how helicases could be used to fulfil a similar role in DNA nanotechnology and selectively increase the off‐rates of DNA hybridization reactions.

As a case study, we investigate how helicase‐driven dehybridization could increase the rate of the primer exchange reaction (PER). PER is a DNA nanotechnology tool that appends new domains with user‐defined sequences onto single‐stranded input strands (primers) (Figure 1a),^[20]^ and is part of a family of templated extension reactions that can recognize inputs of a specific sequence and amplify them. These reactions, which include Polymerase/Exonuclease/Nickase (PEN)^[19]^ circuits, are of interest for molecular and medical applications such as RNA and protein imaging[^24^, ^25^] and for directing active self‐organization.[^26^, ^27^]

PER appends new domains with user‐defined sequences onto single‐stranded input strands (primers) in a four‐step process (Figure 1a).^[20]^ First, a hairpin with a single‐stranded 3′ overhang reversibly binds to the primer (*equilibrium binding*). Then a DNA polymerase extends the primer by copying the template domain on the hairpin (*DNA polymerization*). During this polymerization step, the nascent strand displaces the top strand in the hairpin. Next, the displaced hairpin domain competes for binding to the template domain on the hairpin with the nascent strand in a reversible strand‐displacement reaction (*strand‐displacement*). Finally, the product is reversibly released from the hairpin (*equilibrium release*). PER is done at high polymerase concentrations so that either the reactant‐catalyst binding or the product‐catalyst unbinding, but not the polymerization step is rate‐limiting. PER can extend primers of 10–12 nucleotides in just minutes at 37 °C,^[20]^ but extension of longer or shorter primers is much slower (Supporting Information Figure 2), consistent with the notion that the binding strength between reactant and catalyst can be neither too weak nor too strong (Figure 1b).

We show how to expedite PER in the strong‐binding regime by coupling the reaction to active dehybridization of DNA by ATP‐dependent helicases. Specifically, we use Rep‐X, which is an engineered “super” helicase that has a higher unwinding activity than its wild type counterpart Rep and selectively targets DNA duplexes with an 3′ single‐stranded overhang.^[29]^ This selectivity facilitates the design of catalytic reactions where the product‐catalyst duplex is separated by Rep‐X, but the reactant‐catalyst complex does not have a 3′ overhang—as is the case for PER—and is protected. As a result, a helicase can more rapidly remove a reaction product, speeding up the rate of the last step of a catalytic process without slowing down the initial substrate–catalyst binding step. We will show that Rep‐X selectively unwinds product‐catalyst but not primer‐catalyst duplexes, increases the product's off‐rate, and thus speeds up the reaction in the strong binding regime.

To develop this expedited Primer Exchange Reaction, we first establish an analytical model that predicts the dependence of the PER rate on reaction temperature and primer length, and captures why PER occurs quickly only in a narrow range of primer lengths for a given temperature. We then use this model to predict the effect of helicase activity on the PER rate. Next, we test helicase activity on DNA complexes with and without a 3′ overhang. Finally, we measure the PER rate in the presence of helicase and show that it agrees well with our prediction, demonstrating how helicases can be used as a predictable tool in DNA nanotechnology.

## Results and Discussion

### Analytical Model of PER Rate

**Figure 2:**
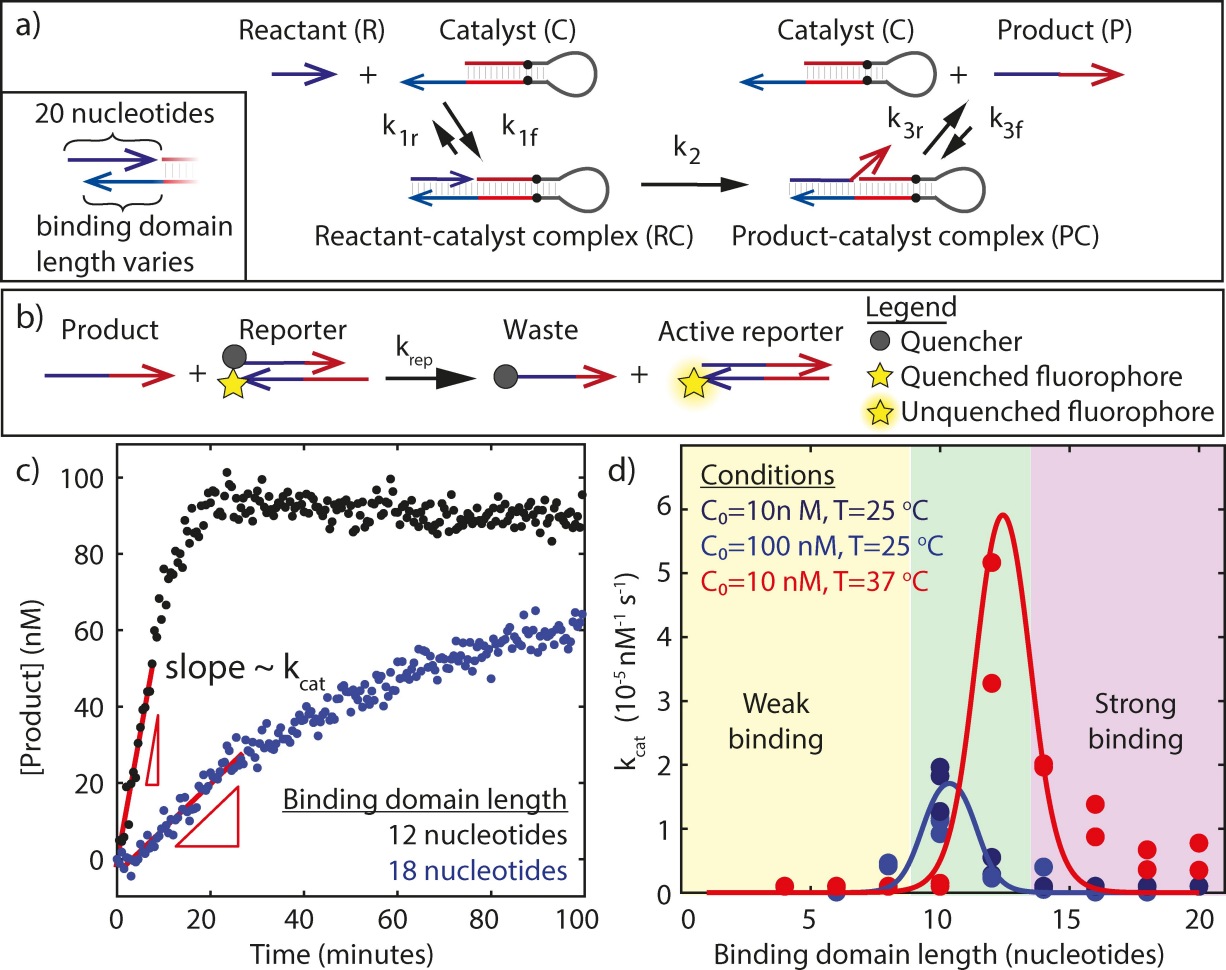
a) Overview of the PER reaction. The reactant or primer (blue) is a 20 nucleotide single‐stranded DNA. It binds to the blue single‐stranded binding domain on the catalytic hairpin. This binding domain can vary in length from 4 to 20 nucleotides. Black dots represent a stop sequence for DNA polymerase. Dark and light red and dark and light blue strands each have complementary sequences. b) Reporting scheme for measuring the output of PER. The PER product reacts with the reporter via a 6 nucleotide toehold strand‐displacement reaction. This reaction separates the quencher‐labeled strand from the fluorophore‐labeled strand in the reporter complex and produces a fluorescent signal proportional to the product strand concentration. c) Measured PER product concentration as a function of time. The turnover frequency *k*_cat_ was determined by dividing the initial slope by *R*_0_ and *C*_0_. The experiment was conducted at 37 °C using catalyst hairpins with binding domains 12 (black) and 18 (blue) nucleotides in length. Both binding domains only contained A's and T's. *C*_0_=10 nM and *R*_0_=100 nM. d) The turnover frequency *k*_cat_ as a function of binding domain length for a range of experimental conditions. Dark and light blue dots represent experiments conducted at 25 °C and red dots represent experiments conducted at 37 °C. *C*_0_=10 nM in all experiments except the light blue ones, where *C*_0_=100 nM. *R*_0_ is either 100 nM or 200 nM (see Supplementary Methods for details). The curves represent fits of Equation (3) to the data with *k*_2_ as the only adjustable parameter. We find that *k*_2_≈0.002 at 25 °C and *k*_2_≈0.008 at 37 °C. In the model we use *R*_0_=100 nM.

To understand how the PER rate depends on the binding energy between the reactant/product and catalyst strands, we develop a simple analytical model that combines features of the three‐step model for toehold‐mediated strand displacement reactions^[30]^ and from Michaelis–Menten kinetics.^[31]^ In our model, the primer binds the hairpin during *equilibrium binding* with forward and reverse (or on‐ and off‐) rate constants *k*_1*f*_ and *k*_1*r*_ respectively (Figure 2a). We model the *DNA polymerization* and *strand‐displacement* steps as a single, irreversible reaction with an effective rate constant, *k*_2_ (Figure 2a). Finally, the product is released from the hairpin during *equilibrium release* with forward and reverse rate constants *k*_3*f*_ and *k*_3*r*_ respectively (Figure 2a).

When PER proceeds at steady‐state and there is much more reactant than catalyst, the reaction can be modeled as a process in which only the reactant concentration $\left[R\right]$ and the product concentration $\left[P\right]$ change over time, i.e. the concentrations of the unoccupied catalyst $\left[C\right]$ , the catalyst‐reactant complex $\left[RC\right]$ , and the catalyst‐product complex $\left[PC\right]$ remain constant. This model is analogous to Michaelis–Menten kinetics for enzymatic reactions where the catalyst strand takes the role of the enzyme,^[31]^ except that we consider the conversion of reactant to product and the release of the product from the catalyst to be two separate steps, analogous to the three‐step‐model for DNA strand‐displacement reactions.^[17]^ Under these assumptions the differential equations governing the reaction are:

(1) $$\frac{d\left[RC\right]}{dt}=k_{1f}\left[R\right]\left[C\right]-k_{1r}\left[RC\right]-k_{2}\left[RC\right]=0,$$

(2) $$\frac{d\left[PC\right]}{dt}=k_{3f}\left[P\right]\left[C\right]-k_{3r}\left[PC\right]+k_{2}\left[RC\right]=0.$$

Assuming that the reactant and product bind equally strongly to the catalyst, with equilibrium constant *K* ($k_{1f}=k_{3f}\equiv k_{f}$ , $k_{1r}=k_{3r}\equiv k_{r}$ , and $K=k_{f}/k_{r}$ ), and that the reactant concentration is much larger than the catalyst concentration, we can solve Equations 1 and 2 at steady‐state to find that the reactant is consumed as $\left[R\right]\left(t\right)=R_{0}\exp\left(-t/\tau \right)$ , where the reaction half‐time *τ* is given by (see Supporting Information Discussion 1 for derivation)

(3) $$\tau =\left(\frac{1}{k_{2}}+\frac{K}{k_{f}}\right)\left(\frac{R_{0}}{C_{0}}+\frac{1}{KC_{0}}\right).$$

We assume *kf* is a standard hybridization rate between two short DNA strands, reflecting previous findings that this rate of hybridization is not strongly dependent on sequence length or base composition for 10–100 nucleotide reactants. Equation (3) shows that in the limit of very strong binding between catalyst and reactant (large *K*: $\tau \approx \frac{K}{k_{f}}\frac{R_{0}}{C_{0}}$ ), *τ* is proportional to *K*, whereas in the limit of weak binding (small *K*: $\tau =\frac{1}{k_{2}}\frac{1}{KC_{0}}$ ), *τ* is proportional to 1/K . In both cases *τ* is large and the reaction is slow. Only at intermediate binding energy—$K=\sqrt{\frac{1}{R_{0}}\frac{k_{f}}{k_{2}}}$ —does *τ* have a minimum value that corresponds to a peak in reaction rate.

### PER Rate Dependency on Binding Domain Length

To check that our model captures the essential features of PER, we next measure the reaction rate as a function of the binding energy between reactant and catalyst. In our experiments we vary temperature and the length of the binding domain on the catalytic hairpin as control parameters to tune this binding energy. We relate *τ*, the typical reaction half‐time, to the domain length noting that the equilibrium constant depends on the free energy of hybridization between the primer and catalyst, $K=\exp\left[-\Delta G^{o}/k_{B}T\right]$ . The $\Delta G^{o}$ of hybridization is proportional to the length of the complementary domain between the catalyst and the reactant and can be calculated using the nearest‐neighbour model as the sum of the free energies of each of the pairs of hybridized bases.^[15]^ We use that $k_{f}\approx$ 3×10^6^ M^−1^ s^−1^.^[17]^ In our experiments *C*_0_ is either 10 nM or 100 nM and *R*_0_ is either 100 nM or 200 nM. The only unknown parameter in the model is *k*_2_, the polymerization rate of Bst Large Fragment Polymerase, which Deng et al. measured to be around 10^−3^ s^−1^.^[32]^ Using these input parameters, Equation (3) predicts that the reaction rate is maximal for 10‐nucleotide primers at 25 °C and for 12‐nucleotide primers at 37 °C and that shorter or longer primers lead to slower reactions.

To measure the concentration of product over time, we use the reporting scheme outlined in Figure 2b. The reporter was designed to have a 6‐base overhang so that the rate constant for the reaction between product and reporter $k_{\mathrm{rep}}\approx$ 10^−3^ nM^−1^ s^−1^ $\gg k_{\mathrm{cat}}$ .^[30]^ Moreover, the waste strand in the reporter duplex binds to the catalyst with the same strength as the product so that the downstream reaction of product with reporter shouldn't shift the equilibrium. We verified that these conditions are met and the reporter complex doesn't affect reaction rate by measuring the product concentration over time both based on fluorescence measurements using the reporter in Figure 2b and directly using gel electrophoresis, and we found good agreement (Supporting Information Figure 3 and 4).

Figure 2c shows product formation over time for a typical PER experiment (see Supporting Information Figure 5 for conversion from fluorescence to concentration). We measured the initial rate at which product strand is formed (Figure 2c) and divided it by *C*_0_ and *R*_0_ to obtain *k*_cat_. The reaction rate scales linearly with catalyst concentration, so *k*_cat_ is a measure for reaction rate that is independent of catalyst concentration and allows comparison of experiments with varying catalysts concentrations. It is related to *τ* as $1/\tau \approx C_{0}k_{\mathrm{cat}}$ for t≪τ .

Our derivation of *τ* (Supporting Information Disc. 2) assumes that the reaction rate is either limited by reactant binding or by product release, and that the polymerization itself is not rate‐limiting in PER. Consistent with that assumption we found that decreasing the concentration of DNA polymerase 10‐fold does not decrease the reaction rate (Supporting Information Figure 6).

Figure 2d shows both the predicted and measured reaction rates as function of the binding domain length at 25 °C and 37 °C. At 25 °C, the experimentally observed peak in reaction rate lies at around 10 nucleotides and at 37 °C the peak is around 12 nucleotides, in agreement with our predictions. Values of *k*_2_=2×10^−3^ s^−1^ at 25 °C and *k*_2_=8×10^−3^ s^−1^ at 37 °C produce a close correspondence between the model and the experiment (see Supporting Information Discussion 2 for a list of the used parameters). These polymerization rates are consistent with the ones measured by Deng et al. who also found that the rate increases with temperature.^[32]^

Despite an overall good agreement, the measured rates for long binding domains are higher than our predicted values, which can be vanishingly small. One reason for this may be that low DNA reaction rates can be difficult to measure precisely in bulk because some DNA strand may have sequence errors that allow them to react faster^[33]^ and our strands are unpurified after solid‐state synthesis so a fraction of strands is expected to contain deletions.

The agreement between the prediction of Equation (3) and our experimental findings shown in Figure 2 supports the idea that the PER is fast only when the occupancy time of the product is within a particular range. The occupancy time must be short enough that the product detaches, allowing the reaction to complete, but not so short that the reactant, which has the same occupancy time, cannot bind long enough for the polymerase to extend it while it is bound. At a given temperature, these occupancy times depend exponentially on the hybridization energy, meaning that PER is only efficient for sequences in a very narrow range of energies. Next, we ask whether the PER rate can be sped up by using an enzyme that separates DNA duplex regions at a rate independent of the hybridization energy. In this case, when the hybridization is fast and binding strong, product‐catalyst separation would occur primarily because of enzymatically‐driven separation, decoupling the PER rate from the hybridization energy. To test this idea, we next explore how the addition of an ATP‐dependent helicase separates DNA complexes at a sequence length‐independent rate and thereby enables a wider range of lengths for PER.

### Predicted Effect of Helicase on PER

Helicases, a class of ATP‐dependent enzymes that unwind double stranded DNA, can help expedite PER by increasing the product off‐rate beyond the equilibrium rate. We use the engineered helicase Rep‐X, which selectively targets complexes with a single‐stranded 3′ overhang. This selectivity is a desirable feature in PER because it causes Rep‐X to remove product from the catalyst without affecting the residence time of the reactant on the catalyst. While in the ideal case Rep‐X only unwinds complexes with 3′ overhangs, Rep‐X also unwinds double‐stranded DNA without 3′ overhangs, albeit at a lower rate.^[29]^ We will measure this selectivity in Section 2.4.

**Figure 3:**
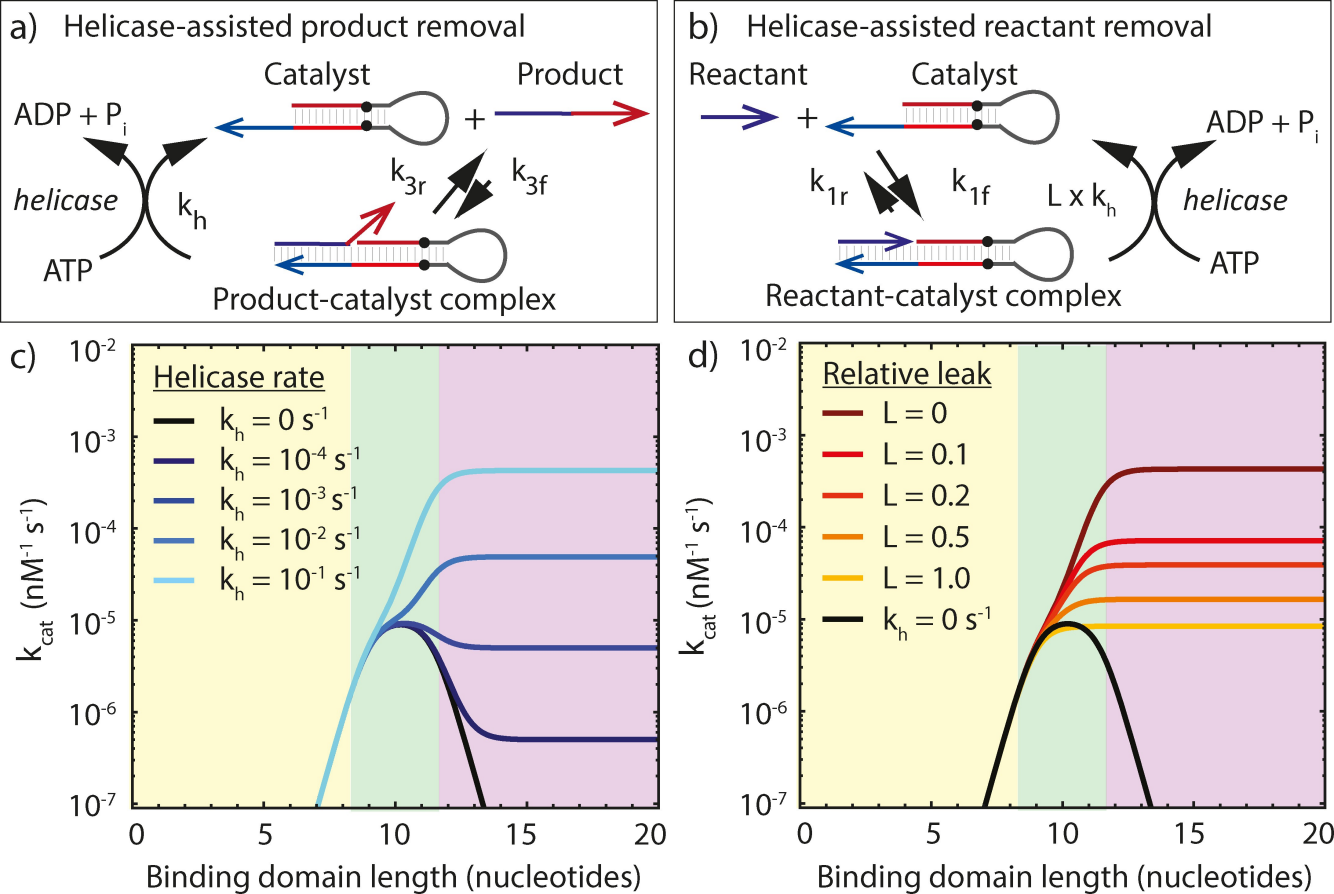
a) Schematics of the intended reaction in which helicase removes product from the catalyst strand (top). b) Schematics of the leak reaction in which helicase removes unreacted primer from the catalyst strand. c) Turnover frequency versus binding domain length for a range of helicase‐assisted product removal rates, as predicted by Equation (6). A higher helicase rate results in a faster reaction for long binding domain lengths. The rate at short binding domain lengths is unaffected. d) Turnover frequency versus binding domain length for a range of leak rates using *k*_h_=0.1 s^−1^. Even a nonselective helicase (*L*=1) expedites PER for large binding lengths, but not as effectively as selective helicases. The peak rate (at optimal binding domain length) can only be increased by a selective helicase. In all the calculations *C*_0_=100 nM, *R*_0_=200 nM, *T*=25 °C, and *k*_2_=2×10^−3^ s^−1^.

To quantify how Rep‐X affects the PER rate, we include terms in Equations (1) and (2) to account for the unwinding of the product‐catalyst complex at rate *k_h_* (see Figure 3a) and the unintended removal of the reactant from the catalyst with a leak rate $k_{l}=L\times k_{h}$ (see Figure 3b):

(4) $$\frac{d\left[PC\right]}{dt}=k_{3f}\left[P\right]\left[C\right]-\left(k_{3r}+k_{h}\right)\left[PC\right]+k_{2}\left[RC\right]=0.$$

(5) $$\frac{d\left[RC\right]}{dt}=k_{1f}\left[R\right]\left[C\right]-\left(k_{1r}+k_{2}+k_{l}\right)\left[RC\right]=0.$$

Here, *k_h_* is a rate constant with units *s* ^–1^ and the leak parameter *L* is a dimensionless constant between 1 and 0 that captures the relative rate at which Rep‐X unwinds complexes without 3′ overhangs compared to complexes with 3′ overhangs. *L* is 0 for a leak‐free reaction and is 1 if the 3′ overhang makes no difference. We follow the same derivation as outlined in section 2.1, but have to make an additional simplification (details in Supporting Information Discussion 1) to arrive at an analytical expression for the reaction timescale in the presence of helicase:

(6) $$\tau =\left(\frac{1}{k_{2}}+\frac{K}{k_{f}}\left(1+\frac{k_{l}}{k_{2}}\right)\right)\left(\frac{\frac{R_{0}}{C_{0}}}{1+K\frac{k_{h}}{k_{f}}}+\frac{1}{KC_{0}}\right)$$

Equation (6) shows that the addition of helicase introduced a second off‐rate, *k_h_* (and *k_l_* which is proportional to *k_h_*), which is similar to *k_r_*, but not related to the on‐rate via the equilibrium constant. Note that if $k_{h}=0$ , Equation (6) equals the expression in Equation (3) in which we did not consider a helicase, as it should.

Figure 3c depicts the predicted turnover frequency $k_{\mathrm{cat}}=\frac{1}{C_{0}\tau }$ as a function of binding domain length for varying helicase rates, considering a perfectly selective helicase (L=0 ). It shows that the reaction rate is affected by helicase only in the strong binding regime. Before the peak, the reaction rate is limited by the on‐rate of reactant and unaffected by the addition of helicase. After the peak, the reaction rate is limited by the product off‐rate and increases due to the addition of helicase. The increase only manifests in the regime where $k_{h}>k_{r}$ (If $k_{h}\ll k_{r}$ , then $1+K\frac{k_{h}}{k_{f}}\to 1$ and $\frac{K}{k_{f}}\frac{k_{l}}{k_{2}}\to 0$ so that Equation (6) reduces to Equation (3)).

Figure 3d shows the influence of the unintended helicase‐assisted removal of the reactant from that catalyst (with rate $L\times k_{h}$ ) on the PER rate. Notably, it shows that a selective helicase is not required to expedite PER, but higher selectivity results in a larger rate increase. Taken together, these findings show that a helicase could dramatically reduce the PER rate's sensitivity to domain length in the strong binding regime, even if it is not entirely selective.

### Helicase Unwinding Rate and Leak

**Figure 4:**
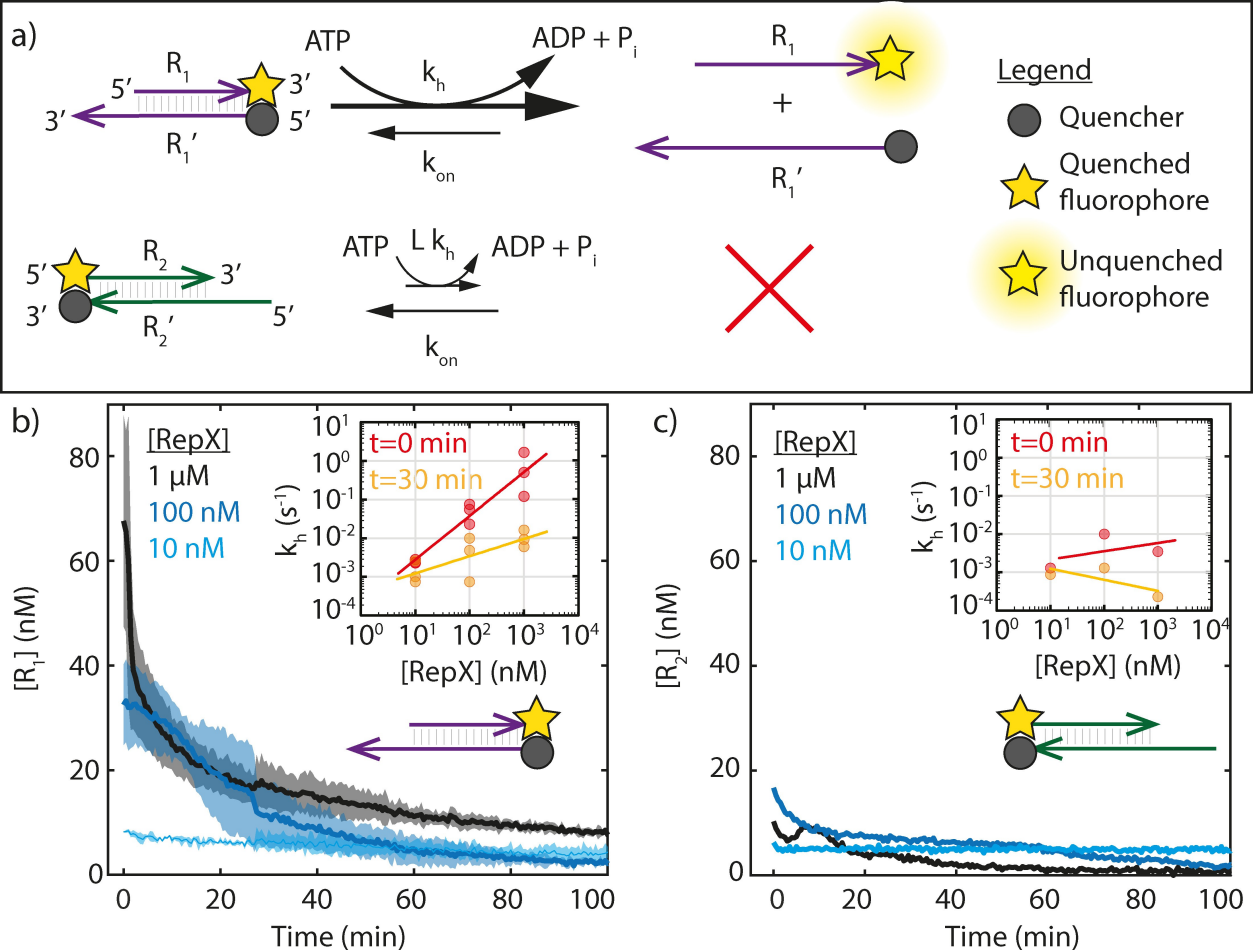
a) Overview of the experiment to test Rep‐X helicase performance. Strands of the same color have complementary sequences and hybridize. The dark yellow stars indicate a quenched FAM fluorophore, the gray sphere indicates the Iowa Black quencher. Measured fluorescence increases with increasing concentration of *R*_1_ or *R*_2_. Rep‐X helicase preferentially unwinds complexes with a 3′ (rather than 5′) single‐stranded overhangs. b) Concentration of free reporter strand *R*_1_ in a sample with the reporter complex *R*_1_:*R*′_1_ directly after the addition of Rep‐X helicase and 1 mm ATP. We calculated [*R*_1_] by comparing the fluorescent signal during the experiment with the fluorescent signal of a sample with separate fluorophore and quencher strands. Shaded area indicates the standard deviation based on three independent experiments. The inset shows the inferred *k*_h_ for a range of Rep‐X concentrations directly after mixing and after half an hour. The decrease in helicase activity over time is due to ATP depletion (Supporting Information Figure 7 and 8). c) Same data as in b) but for a reporter with a 5′ overhang. The helicase‐mediated unwinding rate is substantially lower for *R*_2_ : *R*′_2_ than for *R*_1_ : *R*′_1_

To predict the effect of Rep‐X on the PER rate, we measure *k_h_* and the leak rate of Rep‐X using the two reporter complexes shown in Figure 4a. These complexes have identical sequences except that one of the two reporters, $R_{1}:R_{1}{}^{'}$ (depicted in purple), has a 3′ overhang whereas the other, $R_{2}:R_{2}{}^{'}$ (depicted in green), has a 5’ overhang. When the reporter complexes are hybridized, the fluorophore on one reporter's strand is in close proximity to a quencher on the other, dampening the fluorescent signal. In equilibrium, the spontaneous off‐rate of the $R_{1}:R_{1}{}^{'}$ complex is negligible and all *R*_1_ is hybridized to $R_{1}{}^{'}$ . The fluorescent signal thus indicates the concentration of unhybridized *R*_1_, from which we can calculate *k_h_*.

Figure 4b shows the concentration of *R*_1_ as a function of time, beginning directly after the addition of Rep‐X helicase and ATP to a solution of $R_{1}:R_{1}{}^{'}$ complex. Initially, most of the 100 nm reporter complex was unhybridized, indicating high Rep‐X activity. Over time, [*R*_1_] decreased, suggesting that the Rep‐X unwinding rate decreased over time. We found that this decrease is due to ATP depletion, as adding additional ATP causes the fluorescence signal to increase and subsequently decay again (Supporting Information Figure 7) and higher ATP concentrations result in slower decays (Supporting Information Figure 8).

We used the measurements in Figure 4b to obtain an order of magnitude estimate of the helicase rate by noting that the non‐zero concentration $\left[R_{1}\right]$ is due to a competition between the helicase‐mediated off‐rate *k_h_* and the on‐rate *k_f_*. The binding domain of *R*_1_ to $R_{1}{}^{'}$ is 15 nucleotides, so the equilibrium off‐rate *k*_r_—i.e. the off‐rate in absence of helicase—is negligible and in equilibrium $\left[R_{1}\right]$ should be near zero. We thus calculate the *k_h_* values at the three Rep‐X concentrations tested at times t=0 and t=30 minutes from [*R*_1_] at those times using $k_{h}=k_{f}\frac{\left[R_{1}\right]\left[R_{1}{}^{'}\right]}{\left[R_{1}:R_{1}{}^{'}\right]}=k_{f}\frac{{\left[R_{1}\right]}^{2}}{[R_{1}:R_{1}{}^{'}{]}_{0}-[R_{1}]}$ , still assuming $k_{f}=$ 3×10^6^ m s^−1^.

The measured values of *k_h_* are shown in the inset of Figure 4b, which show that *k_h_* increases with Rep‐X concentration. After 30 minutes *k_h_* is smaller for all tested Rep‐X concentrations than it was at time 0. The difference in the rates at these two times also increases as Rep‐X concentration does.

Next, we estimate the leak of Rep‐X helicase—that is the relative rate of unwinding of complexes without a 3′ overhang—by comparing the amount of unbound reporter strand in the experiment containing the purple complex with a 3′ overhang to the experiment containing the green complex with a 5′ overhang, shown in Figure 4c. Interestingly, the leak reaction rate appears to depend only weakly on the Rep‐X concentration. As a consequence, the leak is approximately 1 % for 1 μm Rep‐X but close to 10 % for 100 nM Rep‐X. The leak reaction is likely due to fraying at the blunt end of the *R*_2_ complex, resulting in temporary single stranded 3′ overhangs that are substrates for Rep‐X.

Based on these measurements of Rep‐X's DNA unwinding performance, we can refine our prediction of whether Rep‐X will speed up PER and by how much. We found that the 100 nM Rep‐X resulted in 10^−1^ s^−1^ $<k_{h}<$ 10^−3^ s^−1^ in the 10 to 30 minute window, in which we expect most of the reaction to complete. At those experimental conditions we find that the leak rate is on the order of 10 % which should reduce the efficacy of helicase on expediting PER slightly, as shown in Figure 3d. Using those values we expect the PER rate to be unaffected by Rep‐X in the weak binding regime (0–10 nucleotides), but sped up by at least an order of magnitude in the strong binding regime (10–20 nucleotides) as shown in Figure 3c.

This predicted speed‐up in reaction rate does not come freely and requires the consumption of ATP. As an aside, we quantify the rate of fuel consumption based on the rate decay due to ATP depletion shown in Figure 4b. An exponential fit to the data for 100 nM Rep‐X shows that at those conditions the ATPase rate is on the order of 6×10^−4^ s^−1^ (Supporting Information Figure 9). That means that at the start of the reaction, where [ATP]=1 mM, each Rep‐X molecule consumes 6 ATP molecules per second.

### Helicase Increases PER Rate

Equipped with estimates for the helicase‐directed off‐rate and relative leak of Rep‐X helicase, we moved on to test the prediction that Rep‐X can increase the PER rate in the strong binding regime by expediting the off‐rate of the product without affecting the reactant on‐rate.

**Figure 5:**
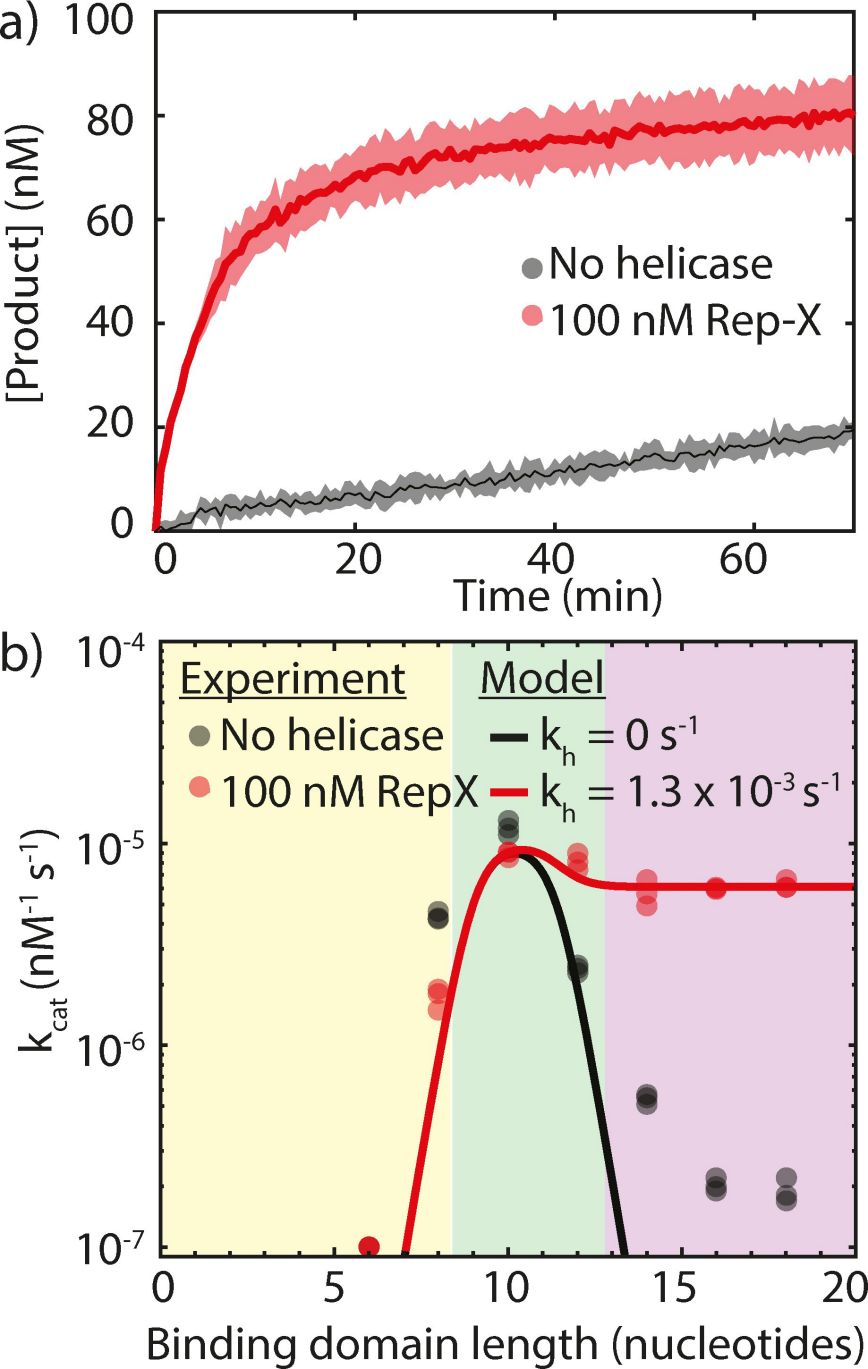
a) Product concentration increase over time in PER reactions with a binding domain length of 16 nucleotides. The black line depicts the average of three experiments in the absence of helicase. The red line depicts the average of three samples containing 100 nM Rep‐X helicase and 1 mm ATP. Shaded areas indicate the standard deviation. In both experiments *C*_0_=100 nM and *R*_0_=200 nM. b) The turnover frequency *k*_cat_ is plotted versus the binding domain length on a semilog plot, resulting in the classical volcano plot. Curves represent the predictions from Equation (3) and (6). The black curve is a fit to the data in absence of helicase with the only adjustable parameter *k*_2_=2×10^−3^. The red curve is a fit to the data in presence of helicase using *k*_2_=2×10^−3^ with *k*_h_=1.3×10^−3^ and *L*=0.1 as the only adjustable parameters. Data points represent individual experiments. Each experiment is done in triplicate.

Figure 5a shows the product concentration as a function of time for a PER reaction with a 16 nucleotide binding domain. In absence of helicase, the strong product‐catalyst bond prevents rapid conversion even with the increased catalyst concentration. The addition of 100 nM Rep‐X and 1 mM of ATP increases the initial rate 30‐fold.

We measured the PER rates for binding domain lengths varying from 6 to 18 nucleotides with and without helicase in triplicate and the results are shown in Figure 5b. The black curve is a fit of Equation (3) to the data without helicase using the value for *k*_2_=2×10^−3^ we found in Figure 2. The red curve is a fit of Equation (6) to the data with helicase, using the same value for *k*_2_ and with the helicase and leak rates as the only adjustable parameters.

The model for the PER rate in presence of helicase matches the experimentally obtained rates well using $k_{h}=$ 1.3×10^−3^ and L=0.1 . This helicase rate is on the low end of the range measured in Figure 4 which is possibly due to a higher overall DNA concentration in the PER experiments (400 nM compared to 100 nM). Notably the hairpins also have a single‐stranded 3′ overhang so a substantial portion of the helicase action is likely wasted on opening hairpins instead of removing product from hairpins.

The presence of helicase limited the yield of the PER reaction (Supporting Information Figure 10, 11), so we studied the reaction at high catalyst concentrations. Under these conditions the quasi‐steady state assumption is longer valid. Surprisingly, our model nonetheless captured the experimentally observed reaction rates as functions of binding energy. This is likely because the main purpose of the model is to capture a transition from reactant binding being the rate‐limiting step to product release being rate limiting. This transition does not rely on the quasi‐steady‐state assumption.

In summary, we showed that Rep‐X‐assisted product removal can expedite PER in the strong binding regime. This finding suggests that Rep‐X could also be used to expedite other multi‐step DNA reactions or reaction networks where the dehybridization step is rate‐limiting. We have identified two design rules to prevent Rep‐X from inadvertently unwinding duplexes that need to remain hybridized for the functionality of these networks: 1) avoid 3′ overhangs where possible and 2) where not possible, modify 3′ overhangs with methylated RNA. We already showed that DNA complexes without 3′ overhangs are protected from Rep‐X‐mediated unwinding. Here we asked if specific complexes with 3′ overhang can also be protected by replacing the DNA 3′ overhang with methylated RNA, because methylated RNA has similar binding properties to DNA and can form Watson–Crick base pairs with DNA strands, but it is not recognized as a substrate by most enzymes. Indeed we found that unwinding rate of Rep‐X is dramatically reduced for complexes with methylated RNA toeholds compared to DNA toeholds (Supporting Information Figure 12). This suggests a design strategy for protecting DNA complexes from unwanted unwinding. It can however not be applied to PER because the 3′ methylated RNA binding domain is also not recognized as a template by the DNA polymerase (Supporting Information Figure 13).

Taken together these data show that a helicase can be used to expedite DNA reactions where the off‐rate is the rate limiting step and that methylated RNA can be used to protect DNA duplexes from unwinding by the helicase.

## Conclusions

Here we asked whether helicases, enzymes that catalyze processive DNA dehybridization, can be incorporated into designed DNA reaction networks to selectively increase off‐rates and thus increase reaction flux. In this case study, we have shown, both theoretically and experimentally, that the PER rate can be increased more than 30‐fold compared to the equilibrium rate in the strong binding regime at the cost of ATP‐hydrolysis, thus circumventing Sabatier's principle. These findings suggest that Rep‐X could also expedite many other DNA reactions where the off‐rates are limiting.[^17^, ^18^, ^21^]

In particular, this method provides a tool to decouple the rate of a step from the sequence length, which can be useful in large reaction networks where the length of a reactant is constrained by conflicting requirements in different steps. This can be useful, for example, when a strand needs to bind strongly in one step but weakly in another, when the length of the sequence is also constrained by the need for high sequence specificity, or where one of the reactants is a biological molecule that is targeted but not designed.

A key advantage of Rep‐X is its propensity to unwind only some duplexes (those with 3′ overhangs) which will allow its use as a sequence‐specific agent within programmed reaction cascades. To direct helicase activity, complexes that should be actively dehybridized in a reaction could present 3′ overhangs, while duplexes whose separation could lead to unwanted interactions could be protected from helicase action by either removing their 3′ overhang or replacing the bases on these overhangs with RNA or methylated RNA.

The concept of active removal of products from catalysts is used broadly—albeit less explicitly—in the polymerase chain reaction (PCR) reaction, where the temperature is oscillated to alternate between strong primer binding and quick product release. Also during the loop‐mediated isothermal amplification of DNA, LAMP, dissipation by a polymerase drives product removal.^[34]^ Milligan and Ellington showed that RecA, an ATP‐dependent DNA‐binding protein, could also speed up DNA reaction cycles.^[23]^ Non‐enzymatic catalytic DNA reactions remove product strands via toehold‐mediated strand displacement, dissipating energy by forming low energy, fully hybridized waste‐products.^[35]^ In this work, we developed a mechanistic understanding of how dissipation can be harnessed that, by its relation to general ideas in chemistry, can be used to drive the design of a wider range of dissipative reaction processes to circumvent kinetic limitations. This framework could conceivably also serve as a foundation for a wider range of incorporation of active agents in DNA networks.

The finding that a dissipative process can be used to expedite a reaction beyond its equilibrium limit imposed by Sabatier's principle raises the question of how much energy needs to be minimally be dissipated to expedite a reaction by a certain amount.^[36]^ We are certainly far from the efficiency limit, because in our experiments with 100 nM Rep‐X, one enzyme hydrolyzed on average 6 ATP per second while only separating on average one base pair.

Theoretical work by Hopfield from 1974 shows the driven release of molecules from a template is required for kinetic proofreading, a process that increases reaction specificity at the cost of energy consumption.^[37]^ The active strand‐separating function of helicases could potentially also be used to increase specificity in DNA reactions via this kinetic‐proofreading method.

## Conflict of interest

The authors declare no conflict of interest.

1

## Supporting information

As a service to our authors and readers, this journal provides supporting information supplied by the authors. Such materials are peer reviewed and may be re‐organized for online delivery, but are not copy‐edited or typeset. Technical support issues arising from supporting information (other than missing files) should be addressed to the authors.

Supporting Information

## Data Availability

The data that support the findings of this study are available from the corresponding author upon reasonable request.
