# Supplementary material for: Catalytic DNA Polymerization Can Be Expedited by Active Product Release
Source: Angew Chem Int Ed Engl. 2022 Apr 20;61(24):e202114581. doi: 10.1002/anie.202114581 (PMC9325435; doi:10.1002/anie.202114581)
Supplement: Supplementary file 1 — Supporting Information [file ANIE-61-0-s001.pdf]

Author Contributions

P.G.M. and R.S. conceived the experiments. P.G.M. performed the experiments and analysis. M.G. produced and purified the helicase. All authors contributed to writing of the paper.
